# Effect of daclizumab high-yield process in patients with highly active relapsing-remitting multiple sclerosis

**DOI:** 10.1007/s00415-013-7196-4

**Published:** 2013-12-29

**Authors:** Gavin Giovannoni, Ernst-Wilhelm Radue, Eva Havrdova, Katherine Riester, Steven Greenberg, Lahar Mehta, Jacob Elkins

**Affiliations:** 1 Queen Mary University of London, Blizard Institute, Barts and The London School of Medicine and Dentistry, 4 Newark Street, London, E1 2AT UK; 2 Medical Image Analysis Center, University Hospital Basel, Basel, Switzerland; 3 Charles University in Prague, Prague, Czech Republic; 4 Biogen Idec, Cambridge, MA USA; 5 AbbVie Biotherapeutics Inc., Redwood City, CA USA

**Keywords:** Relapsing-remitting multiple sclerosis, Drug therapy, Treatment outcome, Safety, Magnetic resonance imaging

## Abstract

Patients with highly active relapsing-remitting multiple sclerosis (RRMS) are at greater risk for disease progression and may respond differently to MS therapeutics than those with less active disease. The current post hoc analysis evaluated the effects of daclizumab high-yield process (DAC HYP) vs. placebo in patients with highly active RRMS in the SELECT study. Highly active RRMS was defined as patients with ≥2 relapses in the year before randomization and ≥1 gadolinium-enhancing (Gd^+^) lesion at baseline. Because results were similar in the DAC HYP dose groups, data from the DAC HYP arms were pooled for analysis. Treatment with DAC HYP resulted in similar effects in highly active (*n* = 88) and less active (*n* = 506) RRMS patients. DAC HYP reduced the annualized relapse rate by 50 % and 51 % in the highly active (*p* = 0.0394) and less active (*p* < 0.0001) groups vs. placebo, respectively (interaction *p* = 0.82). DAC HYP reduced new/newly-enlarging T2 lesions (highly active RRMS 76 % reduction, *p* < 0.0001; less active RRMS 73 % reduction, *p* < 0.0001; interaction *p* = 0.18), the risk of having more Gd^+^ lesions (highly active RRMS 89 % reduction, *p* < 0.0001; less active RRMS 86 % reduction, *p* < 0.0001; interaction *p* = 0.46), and sustained disability progression (highly active RRMS 88 % reduction, *p* = 0.0574; less active RRMS 46 % reduction, *p* = 0.0383; interaction *p* = 0.22) vs. placebo. DAC HYP efficacy was similar across the spectrum of MS disease activity as assessed prior to treatment initiation.

## Introduction

Multiple sclerosis (MS) is characterized by focal demyelination and axonal degeneration within the central nervous system, and subtypes of MS are classified according to the course and stage of disease. Approximately 85 % of patients with MS initially present with a clinically isolated syndrome that evolves into relapsing-remitting MS (RRMS), and eventually secondary progressive MS [1]. However, there is considerable variability in the clinical course of disease within those subtypes. Some patients with RRMS experience slow onset of secondary progressive MS over a period of more than 20 years, whereas others experience more severe symptoms and rapid evolution of MS within a few years of diagnosis [1, 2]. Several studies have shown that the frequency of relapses, the presence of gadolinium-enhancing (Gd^+^) lesions and T2 lesion burden early in the course of MS are predictors for more rapid progression of disability [2–7].

The potential benefits of immunomodulatory therapy in patients with RRMS may be especially important for those with highly active RRMS. Highly active RRMS is sometimes known as rapidly evolving severe RRMS, defined by the European Medicines Agency as patients who have at least two disabling relapses in one year and at least one Gd^+^ lesion on brain magnetic resonance imaging (MRI) or a significant increase in T2 lesion load compared with a previous recent MRI [8, 9]. More frequent relapses and MRI lesion activity early in the course of MS have been associated with greater risk of long-term disability progression [3, 4, 6, 7]. Therefore, it is important to assess the efficacy of new MS therapies in this high-risk subgroup.

Daclizumab is a humanized monoclonal antibody specific for CD25 (the α subunit of the high-affinity interleukin-2 receptor) that is currently being evaluated as a potential treatment for RRMS [10]. The phase II CHOICE study demonstrated that the addition of daclizumab to interferon beta (IFNβ) therapy significantly reduced new or newly-enlarging lesions on brain MRI in patients with RRMS who experienced MS disease activity while on IFNβ monotherapy [11]. More recently, the SELECT study randomized patients with RRMS (*n* = 621) to treatment with subcutaneous daclizumab high-yield process (DAC HYP) 150 mg, DAC HYP 300 mg or placebo every four weeks for 52 weeks [12]. In that study, DAC HYP monotherapy significantly reduced the annualized relapse rate (ARR) and new brain MRI lesion activity, and slowed disability progression in patients with RRMS [12]. The objective of this analysis was to evaluate the efficacy of DAC HYP in patients in the subgroup with highly active RRMS prior to enrollment in the SELECT study.

## Materials and methods

### Study design

Details of the methods of the SELECT study have been published [12]. Briefly, 621 patients with RRMS were randomized to treatment with DAC HYP 150 mg, DAC HYP 300 mg or placebo administered subcutaneously every four weeks for 52 weeks. Eligible patients were 18–55 years of age with RRMS (2005 McDonald criteria) [13] and a baseline Expanded Disability Status Scale (EDSS) score of 0–5.0 [14]. Patients were required to have had at least one confirmed MS relapse within 12 months prior to randomization or one new Gd^+^ lesion on brain MRI performed within six weeks prior to randomization. Patients were excluded if they had primary-progressive, secondary-progressive or progressive-relapsing MS. The primary efficacy endpoint in SELECT was the ARR. Secondary and tertiary outcomes included the effect of DAC HYP vs. placebo on the number of new or newly-enlarging T2-hyperintense lesions at week 52 vs. baseline, the number of Gd^+^ lesions at week 52 vs. baseline and three-month confirmed disability progression. The SELECT study [12] was conducted in compliance with the ethical principles of Good Clinical Practice, according to the International Conference on Harmonisation Harmonised Tripartite Guideline and the ethical principles outlined in the Declaration of Helsinki. Institutional review board/ethics committee approval was obtained at each participating center and patients provided written informed consent at the time of enrollment.

### Patients

Highly active RRMS was defined as at least two relapses in the year prior to randomization and at least one Gd^+^ lesion at baseline, all other patients comprised the less active RRMS subgroup [15, 16]. Efficacy analyses were evaluated in a subset of the intent-to-treat (ITT) population who had nonmissing baseline MRI data. The ITT population included all patients who underwent randomization (except for 21 patients from a single study center who were prospectively excluded from the ITT population owing to detection of systematic misdosing at that center during study monitoring). To be consistent with the efficacy analyses, the safety analyses were evaluated in all randomized patients who had MRI data at baseline. Safety data for all patients in SELECT have been published [12]. Because the efficacy outcomes were similar between the DAC HYP 150 mg and 300 mg groups in SELECT [12], data for both DAC HYP treatment groups were pooled for this analysis.

### Assessments

ARR, time to three-month confirmed disability progression, the risk of having more Gd^+^ lesions at week 52 and the number of new or newly-enlarging T2-hyperintense lesions between weeks 0 and 52 were evaluated for DAC HYP vs. placebo in both the highly active RRMS and less active RRMS subgroups. Relapses were defined as new or recurrent neurologic symptoms that were not associated with fever or infection and which lasted 24 h or more, accompanied by new neurologic findings on assessment by the examining neurologist. An independent committee consisting of MS neurologists blinded to the treatment group adjudicated whether the definition of MS relapse was met. MRI scans were performed for all patients at weeks 24, 36 and 52. Disability progression was defined as at least a 1.0-point increase in EDSS score that was sustained for 12 weeks for patients with a baseline EDSS score of 1.0 or more or at least a 1.5-point increase for patients whose baseline EDSS score was 0. EDSS score was evaluated every 12 weeks and at weeks 20, 52, 60 and 72 as well as at unscheduled relapse visits. Confirmation of disability progression could not take place at a visit when a relapse was occurring.

### Statistical analyses

For each efficacy outcome, the percent reduction and 95 % confidence intervals (CIs) for DAC HYP vs. placebo were estimated in each disease activity subgroup. The ARR was estimated from a negative binomial regression model adjusted for treatment and the number of relapses in the year prior to study entry. The odds ratio for comparison between the DAC HYP and placebo groups of having more Gd^+^ lesions at week 52 was evaluated based on an ordinal logistic regression model adjusted for baseline Gd^+^ lesions and treatment. The mean number of new or newly-enlarging T2-hyperintense lesions between weeks 0 and 52 was estimated from a negative binomial regression model adjusted for treatment and the number of T2 lesions at baseline. The estimated time to progression and proportion of patients with progression was calculated based on the Kaplan–Meier product limit method. The hazard ratio and *p-*value assessing the difference between the treatment groups were estimated from a Cox proportional hazards model controlling for baseline EDSS score (≤2.5 vs. >2.5). *p*-values for the interaction were derived in separate models that also adjusted for baseline, treatment and MS disease activity (highly active RRMS vs. less active RRMS) by treatment variable interaction.

## Results

**Fig. 1:**
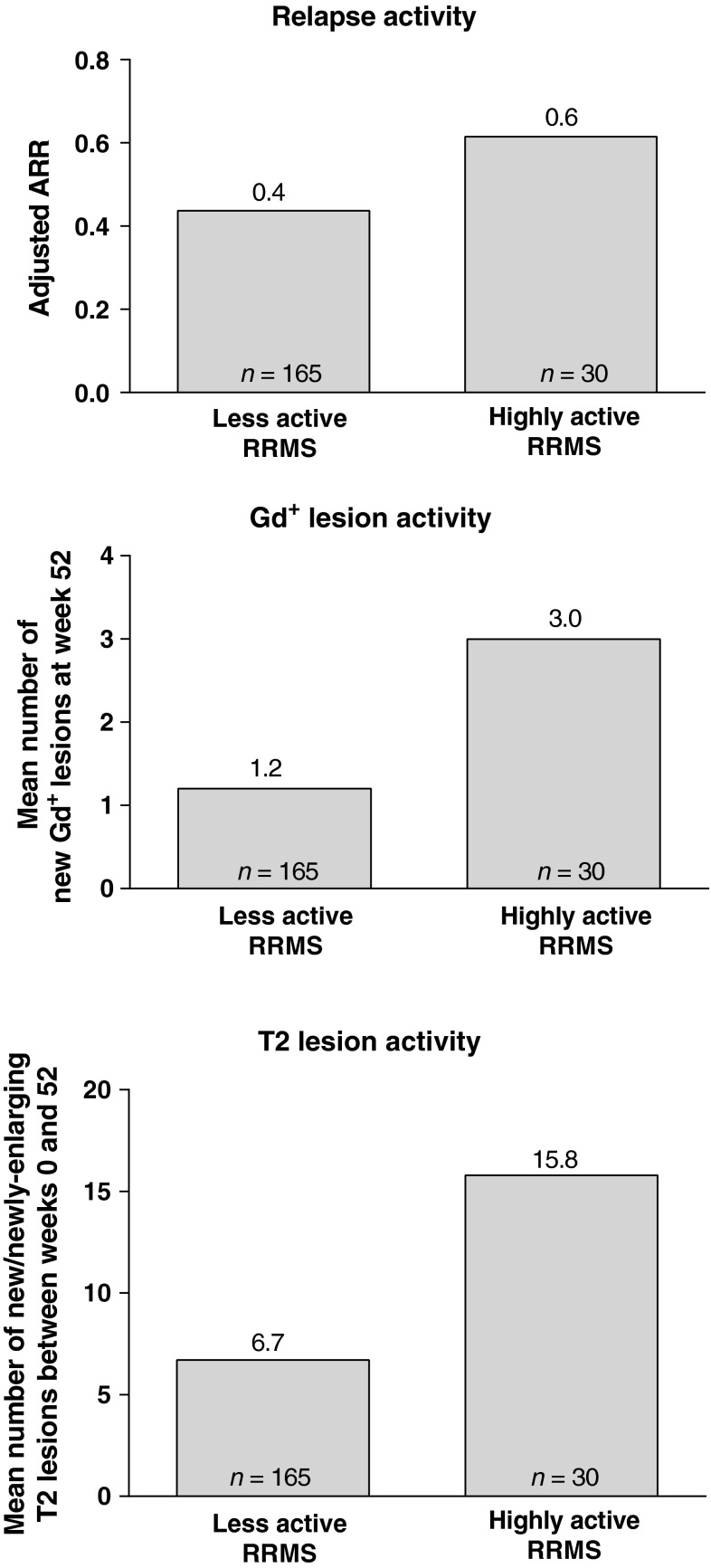
On-study disease activity in patients treated with placebo, by disease activity subgroup. Highly active RRMS was defined as at least two relapses in the year prior to randomization and at least one Gd^+^ lesion at baseline, all other patients comprised the less active RRMS subgroup. *RRMS* relapsing-remitting multiple sclerosis, *ARR* annualized relapse rate, *Gd* ^+^ gadolinium-enhancing

The demographic and clinical characteristics for all randomized patients with baseline MRI data (*n* = 615; Table 1) by disease activity subgroup and treatment are shown Table 1. As would be expected, relapse and MRI lesion activity were higher in patients with highly active RRMS compared with those with less active RRMS. Among the ITT population with MRI data at baseline (*n* = 594), 88 patients met the criteria for highly active RRMS (placebo, *n* = 30 [15 % of placebo]; DAC HYP, *n* = 58 [15 % of pooled DAC HYP]), whereas 506 patients were categorized with less active RRMS (placebo, *n* = 165; pooled DAC HYP, *n* = 341). In patients in the placebo group, on-study ARR was 50 % higher in patients with highly active RRMS compared with those with less active RRMS (Fig. 1). Similarly, in placebo-treated patients, the mean number of new Gd^+^ lesions and the mean number of new or newly-enlarging T2 lesions between weeks 0 and 52 was much greater in patients with highly active RRMS vs. less active RRMS (Fig. 1).

**Table 1:** Demographic and baseline clinical characteristics

	Less active RRMS		Highly active RRMS	
	Placebo (*n* = 173)	DAC HYP (*n* = 351)	Placebo (*n* = 30)	DAC HYP (*n* = 61)
Demographics				
Age, years, mean (SD)	36.5 (9.2)	35.6 (8.7)	37.0 (8.4)	33.0 (9.0)
Sex, female (%)	62	67	67	62
Race, white (%)	97	96	93	98
MS disease characteristics				
No prior approved RRMS therapy (%)^a^	88	84	80	74
Years since MS diagnosis, mean (median)	4.2 (5.3)	4.1 (4.4)	3.7 (5.2)	4.1 (5.5)
Number of relapses in past year, mean (SD)	1.2 (0.5)	1.2 (0.6)	2.2 (0.5)	2.3 (0.7)
Baseline EDSS score, mean (SD)	2.8 (1.2)	2.7 (1.2)	2.5 (1.1)	3.0 (1.3)
MRI brain lesions				
≥1 Gd^+^ lesion (%)	35	34	100	100
Number of Gd^+^ lesions, mean (SD)	1.5 (4.4)	1.3 (2.9)	4.4 (4.4)	4.3 (4.6)
Number of T2-hyperintense lesions, mean (SD)	36.9 (29.9)	37.0 (31.3)	54.2 (40.5)	58.6 (36.8)
Volume of T2-hyperintense lesions, mean (SD)	7,245.7 (8,623.0)	7,656.0 (9,299.8)	12,893.3 (12,214.1)	12,987.1 (11,360.9)

*RRMS* relapsing-remitting multiple sclerosis, *DAC HYP* daclizumab high-yield process, *SD* standard deviation, *MS* multiple sclerosis, *EDSS* Expanded Disability Status Scale, *MRI* magnetic resonance imaging, *Gd* ^*+*^ gadolinium-enhancing

^a^Patients who had no prior use of approved RRMS treatments (interferon beta-1a, interferon beta-1b, natalizumab, glatiramer acetate or mitoxantrone)

**Fig. 2:**
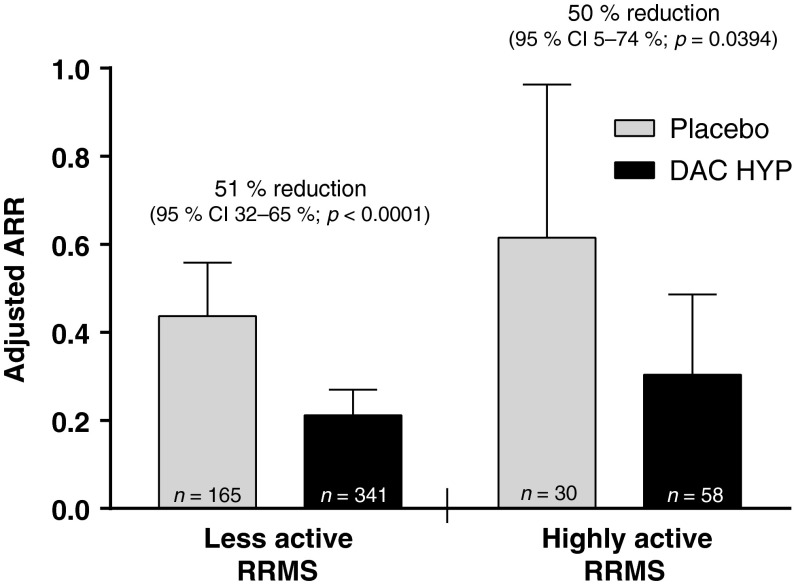
Adjusted ARR and upper 95 % CIs for patients with highly active RRMS or less active RRMS at baseline and treated with DAC HYP or placebo. Highly active RRMS was defined as at least two relapses in the year prior to randomization and at least one gadolinium-enhancing lesion at baseline, all other patients comprised the less active RRMS subgroup. *CI* confidence interval, *DAC HYP* daclizumab high-yield process, *ARR* annualized relapse rate, *RRMS* relapsing-remitting multiple sclerosis

**Fig. 3:**
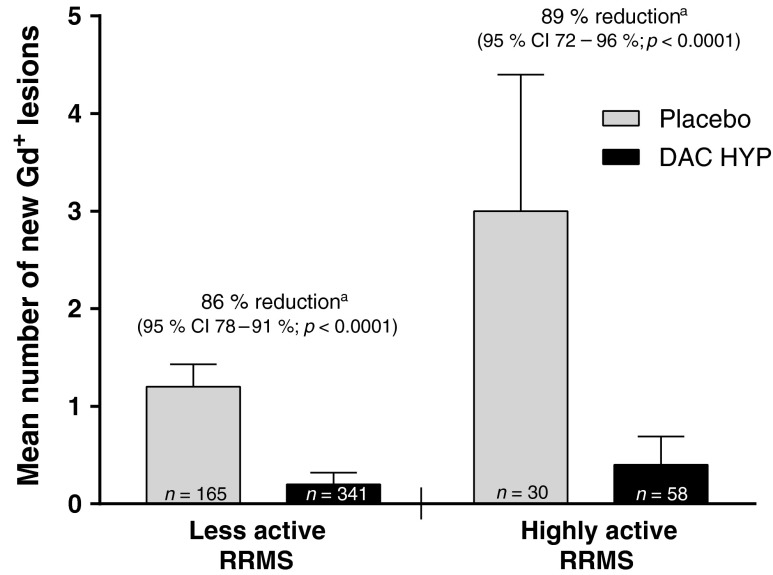
The mean number of new Gd^+^ lesions between weeks 0 and 52 and upper 95 % CIs for patients with highly active RRMS or less active RRMS who were treated with DAC HYP or placebo. Highly active RRMS was defined as at least two relapses in the year prior to randomization and at least one Gd^+^ lesion at baseline, all other patients comprised the less active RRMS subgroup. *p*-values and percentage reduction were estimated from an ordinal logistic regression model adjusted for baseline lesion count in each disease activity subgroup. *CI* confidence interval, *DAC HYP* daclizumab high-yield process, *Gd* ^+^ gadolinium-enhancing, *RRMS* relapsing-remitting multiple sclerosis. ^a^ Percentage reductions represent the reduction over placebo in the risk of having greater Gd^+^ lesion activity

**Fig. 4:**
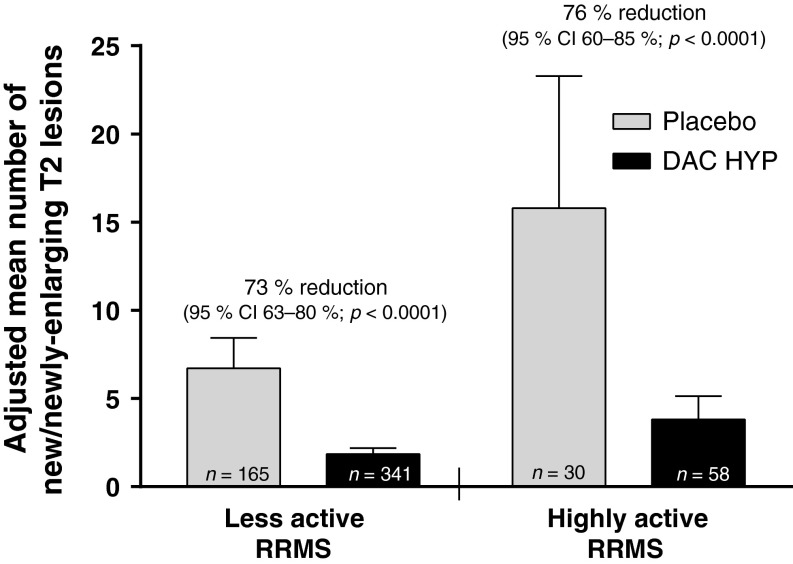
The adjusted mean number of new or newly-enlarging T2-hyperintense lesions between weeks 0 and 52 and upper 95 % CIs for patients with highly active RRMS or less active RRMS who were treated with DAC HYP or placebo. Highly active RRMS was defined as at least two relapses in the year prior to randomization and at least one gadolinium-enhancing lesion at baseline, all other patients comprised the less active RRMS subgroup. *CI* confidence interval, *DAC HYP* daclizumab high-yield process, *RRMS* relapsing-remitting multiple sclerosis

DAC HYP was associated with significant improvements in clinical and radiologic outcomes among patients with highly active RRMS. After one year of DAC HYP treatment, the ARR was reduced by 50 % in patients with highly active RRMS (95 % CI 5–74 %; *p* = 0.0394) and by 51 % in those with less active RRMS (95 % CI 32–65 %; *p* < 0.0001 [interaction *p*-value, *p* = 0.82]; Fig. 2) vs. placebo. DAC HYP treatment had significant effects on brain lesion activity on MRI in both disease activity subgroups. In patients with highly active RRMS, treatment with DAC HYP reduced the risk of having more Gd^+^ lesion activity at week 52 by 89 % (95 % CI 72–96 %; *p* < 0.0001) vs. placebo in the highly active RRMS subgroup and by 86 % (95 % CI 78–91 %; *p* < 0.0001) in the less active RRMS subgroup (interaction *p*-value, *p* = 0.46; Fig. 3). Moreover, the number of new or newly-enlarging T2-hyperintense lesions between weeks 0 and 52 was reduced by 76 % (95 % CI 60–85 %; *p* < 0.0001) in patients with highly active RRMS and by 73 % (95 % CI 63–80 %; *p* < 0.0001) in those with less active RRMS (Fig. 4) for DAC HYP treatment compared with placebo (interaction *p*-value, *p* = 0.18).

**Fig. 5:**
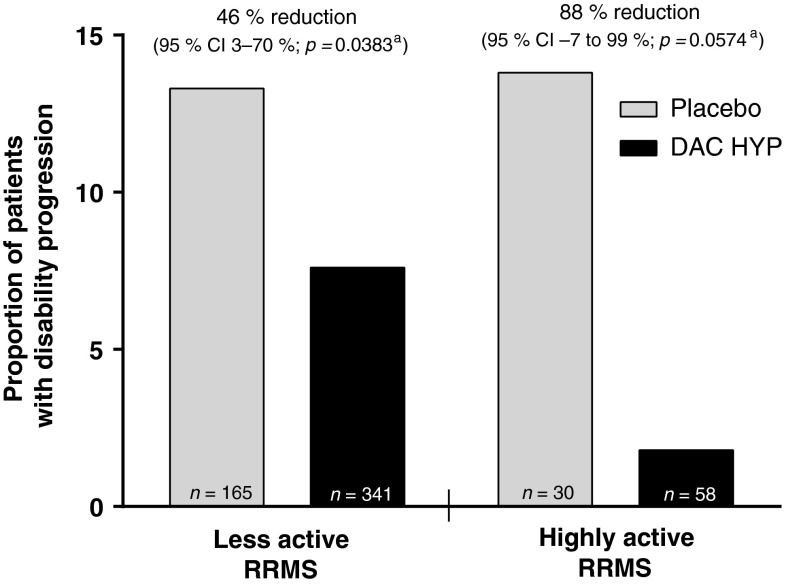
Proportion of patients with three-month confirmed disability progression in patients with highly active RRMS or less active RRMS for DAC HYP vs. placebo. Highly active RRMS was defined as at least two relapses in the year prior to randomization and at least one gadolinium-enhancing lesion at baseline, all other patients comprised the less active RRMS subgroup. *CI* confidence interval, *DAC HYP* daclizumab high-yield process, *RRMS* relapsing-remitting multiple sclerosis. ^a^ *p*-values and percentage reduction for DAC HYP vs. placebo were estimated from a Cox proportional hazards model controlling for baseline EDSS (≤2.5 vs. >2.5) in each disease activity subgroup

Three-month confirmed disability progression was observed in 1.8 % (*n* = 1) of DAC HYP-treated patients vs. 13.8 % (*n* = 4) of placebo-treated patients in the highly active RRMS subgroup and in 7.6 % (*n* = 24) of DAC HYP-treated patients vs. 13.3 % (*n* = 21) of placebo-treated patients in the less active RRMS subgroup. Treatment with DAC HYP reduced the three-month confirmed disability progression by 88 % (95 % CI −7 to 99 %; *p* = 0.0574) in the highly active RRMS subgroup and by 46 % (95 % CI 3–70 %; *p* = 0.0383) in the less active subgroup compared with placebo (interaction *p*-value, *p* = 0.22; Fig. 5).

Similar to what was observed across the treatment groups in the overall patient population in the SELECT study [12], infections, cutaneous events and elevations of liver enzymes, defined as elevations of alanine aminotransferase/aspartate aminotransferase greater than five times the upper limit of normal (5× ULN), occurred more frequently with pooled DAC HYP treatment than with placebo treatment in both the highly active RRMS and less active RRMS subgroups (Table 2). In the current analysis, the proportion of patients with any adverse event (AE) was similar among the disease activity and treatment groups (Table 2). Infections, cutaneous events and elevations of liver enzymes greater than 5× ULN occurred with similar frequency in DAC HYP-treated patients with highly active RRMS and less active RRMS (Table 2). The most common AEs (i.e., occurring in ≥10 % of any disease activity and treatment group), excluding MS relapse, were nasopharyngitis, headache, upper respiratory tract infection and paraesthesias (Table 2).

**Table 2:** AEs by disease activity and treatment group

	Less active RRMS		Highly active RRMS	
	Placebo (*n* = 173)	DAC HYP (*n* = 351)	Placebo (*n* = 30)	DAC HYP (*n* = 61)
Any AE, *n* (%)	136 (79)	258 (74)	25 (83)	49 (80)
Infections	77 (45)	183 (52)	12 (40)	32 (52)
Cutaneous events	25 (14)	72 (21)	2 (7)	11 (18)
Elevation of ALT/AST >5× ULN	1 (<1)	14 (4)	0	3 (5)
Most common AEs, *n* (%)^a,b^				
Nasopharyngitis	27 (16)	50 (14)	4 (13)	10 (16)
Headache	18 (10)	33 (9)	3 (10)	7 (11)
Upper respiratory infection	12 (7)	32 (9)	2 (7)	8 (13)
Paraesthesia	7 (4)	8 (2)	3 (10)	1 (2)

*AE* adverse event, *RRMS* relapse-remitting multiple sclerosis, *DAC HYP* daclizumab high-yield process, *ALT* alanine aminotransferase, *AST* aspartate aminotransferase, *5× ULN* five times the upper limit of normal [12]

^a^Excluding multiple sclerosis relapse

^b^AEs that occurred in ≥10 % of patients in any disease activity and treatment group

## Discussion

In patients with highly active RRMS, treatment with DAC HYP reduced the ARR by 50 % (95 % CI 5–74 %) compared with placebo. This robust effect on relapse rate reduction in patients with highly active RRMS was consistent with the treatment effect of DAC HYP vs. placebo on other key endpoints in this subgroup, such as new T2 lesions (76 % reduction; 95 % CI 60–85 %) and disability progression (88 % reduction; 95 % CI −7 to 99 %). There was no evidence in this analysis that disease activity at baseline modified the effect of DAC HYP on MS activity as treatment effects were similar in both the highly active RRMS and less active RRMS subgroups for all examined endpoints.

There is accumulating evidence for the categorization of patients with highly active RRMS as a clinically meaningful subgroup. Clinical and radiologic evidence of disease activity, including the frequency of relapses [3, 4], a high T2 lesion burden [6] or the presence of Gd^+^ lesions [7] early in the course of MS has been linked to a greater risk of progression of disability over the long term. Additionally, the degree of recovery from the first relapse, time from MS onset to the second neurologic episode and time from MS onset to assignment of an EDSS score of 4.0 have been shown as predictive factors for the onset of irreversible disability [3]. In the current study, differences were observed in on-study relapse activity, the mean number of new Gd^+^ lesions and the mean number of new or newly-enlarging T2 lesions between the highly active RRMS and less active RRMS subgroups of patients in the placebo group (Fig. 1). While patients with highly active RRMS may require an MS treatment with greater efficacy, it is less clear whether in general they are more likely to be refractory to such MS therapies. Previous studies of both natalizumab and fingolimod have also reported maintenance of efficacy in this subgroup of patients [15, 16]. Nevertheless, even when treatment efficacy is preserved on a relative basis, the actual accumulation of inflammatory pathology may be higher in this subgroup of patients with MS over time and confer an increased risk for disease progression.

As highly active RRMS is defined by the frequency of relapses and Gd^+^ lesions, its presence is likely a correlate of the patient’s inflammatory burden of MS. Because daclizumab modulates the immune system by inhibition of high-affinity interleukin-2 signaling [17], we initially hypothesized that DAC HYP may be more effective in patients with highly inflammatory MS. It was notable that treatment efficacy on relapses and MRI lesion activity appeared nearly identical in the subgroups of patients with highly active RRMS and those with less active RRMS. It is plausible that the immunologic effects of daclizumab such as CD56^bright^ natural killer cell expansion [18], inhibition of dendritic cell priming of T cells [19], and a decrease in lymphoid tissue-inducer cells [20] may have efficacy in MS independently of traditional measures of MS-related inflammation. With the caveat that the subgroup sizes were relatively small, it is interesting to note that DAC HYP appeared to have a greater benefit on disability progression in the highly active RRMS subgroup compared with the less active RRMS subgroup. This finding is consistent with the potential for DAC HYP to prevent permanent damage to the central nervous system.

There are limitations to the current analysis. Since the definition of highly active RRMS was based on the patient-reported history of relapse, we could not directly assess whether the relapses had been disabling, as indicated in some definitions of highly active RRMS [8, 9]. Additionally, we could not directly evaluate whether historic relapses occurred while the patient had been treated with a disease-modifying therapy. Thus, we could not determine whether the highly active RRMS subgroup also had evidence of being refractory to other MS treatments. However, clear differences in MS disease activity between placebo-treated patients characterized at baseline as highly active or less active indicated the characterization used in this analysis accurately reflected patients’ risk for incident MS-disease activity (Fig. 1). Finally, because this was a post hoc analysis of the SELECT trial and owing to the relatively small subgroup sample sizes, the results should be confirmed independently.

Since patients with highly active RRMS are at higher risk for disease progression, these patients may have different risk-benefit considerations for MS therapies, and it is important to evaluate the performance of MS treatments in this subgroup of patients. Further refinement of MS subtypes and prognostic markers may improve the ability to individualize therapeutic decision making for this group of patients. The ongoing three-year phase III trial of DAC HYP consisting of 1,800 patients (DECIDE: ClinicalTrials.gov identifier NCT01064401) will further inform on the potential of DAC HYP as a treatment option for this high-risk subgroup of the MS population.

## Study investigators and other participants

*Select study investigators* Czech Republic: Prof. Zdeněk Ambler, Prof. Ivan Rektor, Dr. Radomir Talab, Prof. Petr Kanovsky, Dr. Pavel Stourac, Dr. Denisa Zimova and Dr. Marta Vachova. Germany: Prof. Dr. Bernd C. Kieseier, Dr. Björn Tackenberg, Prof. Dr. Heinz Wiendl, Prof. Dr. Reinhard Hohlfeld, Dr. Klemens Angstwurm, Prof. Dr. Judith Haas, Prof. Dr. Uwe Zettl, Prof. Dr. Florian Stögbauer, Dr. Ralf Linker, Prof. Dr. Andrew Chan and Prof. Dr. Patrick Oschmann. Hungary: Dr. Attila Csányi, Dr. Péter Diószeghy, Dr. János Nikl, Dr. Gyula Pánczel, Dr. Béla Clemens, Dr. Etelka Jófejű, Dr. Attila Valikovics, Dr. István Kondákor, Dr. Dániel Bereczki, Dr. Zsuzsanna Lohner, Prof. Lászlo Csiba, Dr. András Folyovich, Dr. Péter Harcos, Dr. Gabriella Kovács and Dr. Mária Sátori. India: Dr. Rajaram Agarwal, Dr. Pahari Ghosh, Dr. Sangeeta Ravat, Dr. Subhash Mukherjee, Dr. Rustom Wadia, Prof. Kolichana Venkateswarlu, Dr. Meenakshisundaram Umaiorubahan, Prof. Medasari Padma, Dr. Thomas, Dr. A K Meena and Dr. Suresh Kumar. Poland: Dr. Wieslaw Drozdowski, Dr. Waldemar Fryze, Dr. Jan Kochanowicz, Prof. Anna Kaminska, Dr. Krzysztof Selmaj, Dr. Andrzej Szczudlik, Dr. Andrzej Wajgt, Dr. Anna Czlonkowska, Dr. Zbigniew Stelmasiak, Dr. Gabriela Klodowska-Duda, Dr. Janusz Zbrojkiewicz. Romania: Dr. Ovidiu Bajenaru, Dr. Dafin Fior Muresanu and Dr. Mihaela Simu. Russia: Dr. Olga Vorobyeva, Dr. Leonid Zaslavsky, Dr. Sergey Shvarkov, Dr. Miroslav Odinak, Dr. Anna Belova, Dr. Irina Sokolova, Dr. Farit Khabirov, Dr. Natalia Nikolaevna Maslova, Dr. Irina Poverennova, Dr. Nikolay Spirin, Dr. Nadezhda Malkova, Dr. Semen Prokopenko, Dr. Alexey Rozhdestvensky, Dr. Alexei Boiko and Dr. Rim Magzhanov. Turkey: Prof. Sabahattin Saip, Prof. Omer Faruk Turan, Ass. Prof. Serhat Ozkan and Prof. Ayse Sagduyu Kocaman. Ukraine: Dr. Natalia Buchakchyys’ka, Dr. Natalia Lytvynenko, Dr. Borys Palamar, Dr. Tatyana Nehrych, Dr. Natalia Voloshina, Dr. Larisa Sokolova, Prof. Olexandr Kozyolkin, Dr. Olena Moroz, Prof. Valeriy Pashkovskyy, Dr. Elena Statinova, Dr. Tetjana Kobys and Dr. Igor Pasyura. UK: Dr. Clive Hawkins, Dr. Basil Sharrack, Dr. Cris Constantinescu, Dr. John Zajicek, Dr. David Bates and Dr. Eli Silber. Data Safety Monitoring Board: Dr. Volker Limmroth (Chair), Dr. Richard Furie, Dr. Daniel McQuillen, Dr. Raymond Chung, and Dr. Richard Kay. Relapse Adjudication Committee: Dr. Chris Polman, Dr. Ted Phillips, Dr. Paul O’Connor, Dr. Ari Green and Dr. Oliver Lyon-Caen.
